# Supplementary material for: GPR97 triggers inflammatory processes in human neutrophils via a macromolecular complex upstream of PAR2 activation
Source: Nat Commun. 2022 Oct 27;13:6385. doi: 10.1038/s41467-022-34083-1 (PMC9613636; doi:10.1038/s41467-022-34083-1)
Supplement: Supplementary file 1 — Supplementary Information [file 41467_2022_34083_MOESM1_ESM.pdf]

Supplementary Information

GPR97 triggers inflammatory processes in human neutrophils via a macromolecular complex upstream of PAR2 activation

Chu et al. 2022

Supplementary Figure 1: GPR97^{E}-mFc induces neutrophil activation by interacting with a putative GPR97-ligand expressed in a distinct human neutrophil subpopulation.
Supplementary Figure 2: Identification of mPR3 as the GPR97-specific binding partner on human neutrophils.
Supplementary Figure 3: GPR97 is a positive allosteric effector of neutrophil mPR3.
Supplementary Figure 4: Crystallographic studies of GPR97-ECR.
Supplementary Figure 5: The NTD is stabilised by hydrophobic interactions conserved in all GPR97 orthologs, but not found in other GAIN domains.
Supplementary Figure 6: Mapping of the mPR3-binding regions in GPR97-ECR.
Supplementary Figure 7: Expressional regulation of GPR97 and PAR2 in neutrophils.
Supplementary Figure 8: FcR-dependent up-regulated expression of GPR97 and PAR2 in neutrophils.
Supplementary Figure 9: Comparison of neutrophil activation phenotypes triggered by PAR2-specific agonists and GPR97^{E}-mFc.
Supplementary Figure 10: FACS strategy of gating human neutrophil leukocytes from blood samples.
Supplementary Table 1: Antibody list
Supplementary Table 2: Cell line list
Supplementary Table 3: Primer list
Supplementary Table 4: Crystallographic statistics of GPR97-ECR.

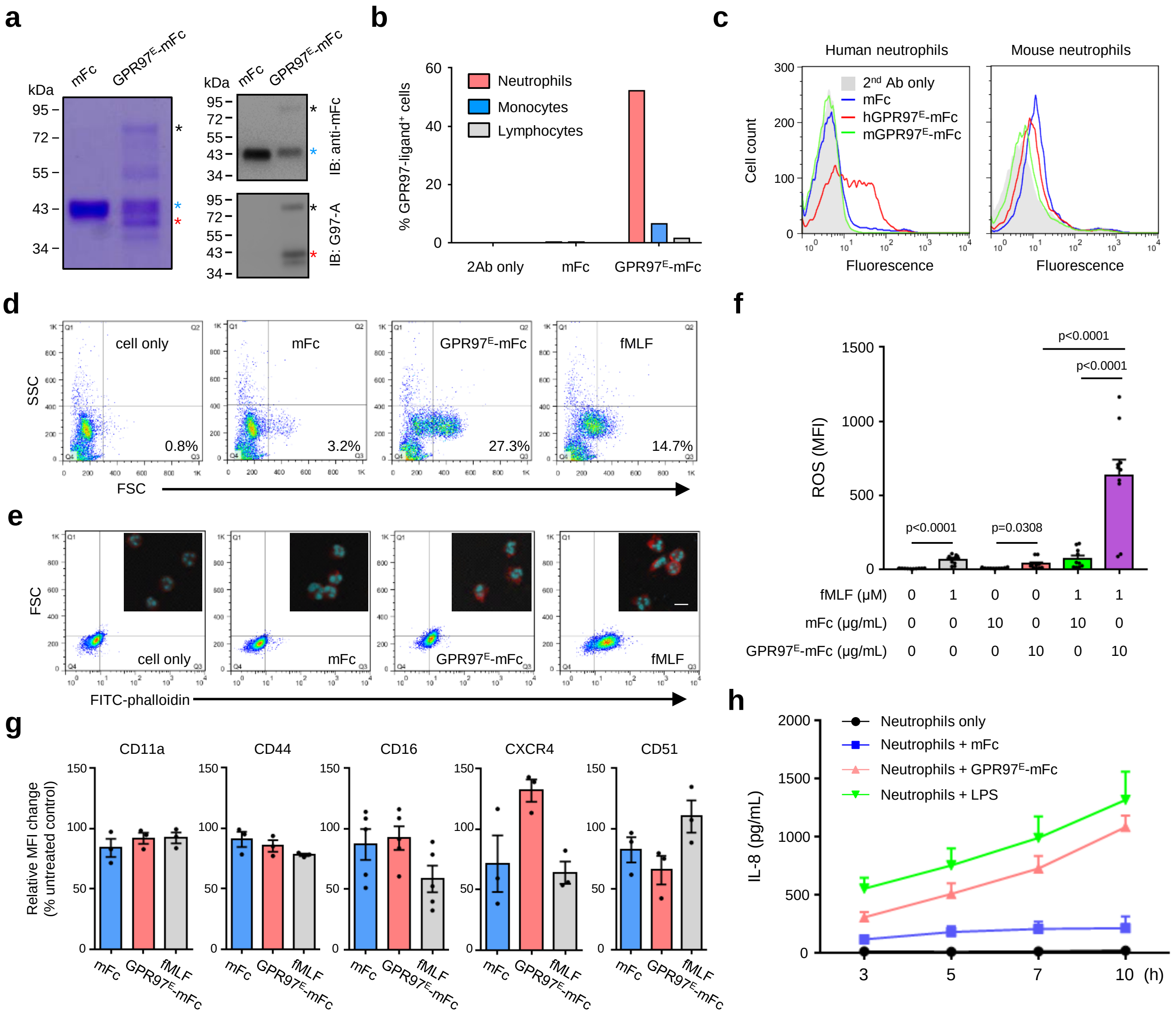
a
kDa
mFc
GPR97E-mFc
95
72
55
43
34
IB: anti-mFc
IB: G97-A
b
% GPR97-ligand+ cells
Neutrophils
Monocytes
Lymphocytes
60
40
20
0
2Ab only
mFc
GPR97E-mFc
c
Human neutrophils
Mouse neutrophils
Cell count
2nd Ab only
mFc
hGPR97E-mFc
mGPR97E-mFc
300
200
100
0
Fluorescence
d
SSC
FSC
cell only
mFc
GPR97E-mFc
fMLF
0.8%
3.2%
27.3%
14.7%
e
FSC
FITC-phalloidin
cell only
mFc
GPR97E-mFc
fMLF
f
ROS (MFI)
1500
1000
500
0
p<0.0001
p<0.0001
p<0.0001
p=0.0308
fMLF (μM) 0 1 0 0 1 1
mFc (μg/mL) 0 0 10 0 10 0
GPR97E-mFc (μg/mL) 0 0 0 10 0 10
g
Relative MFI change (% untreated control)
CD11a
CD44
CD16
CXCR4
CD51
150
100
50
0
mFc
GPR97E-mFc
fMLF
h
IL-8 (pg/mL)
2000
1500
1000
500
0
Neutrophils only
Neutrophils + mFc
Neutrophils + GPR97E-mFc
Neutrophils + LPS
3
5
7
10 (h)

Supplementary Fig. 1 GPR97^E-mFc induces neutrophil activation by interacting with a putative GPR97-ligand expressed in a distinct human neutrophil subpopulation. **a.** Biochemical analyses of GPR97^E-mFc protein by coomassie blue staining in SDS-PAGE gels (left panel) and by western blotting (right panels). Left panel: the molecular weight of GPR97-NTF is ~40 kDa (indicated by red asterisk), similar to the size of mFc (blue asterisk). A minor fraction of ~82 kDa un-cleaved GPR97^E-mFc (black asterisk) was also expressed. The mFc-only protein was included as a control. Right panels: Western blot analyses of GPR97^E-mFc using Abs specific to mFc (top) and to GPR97-ECR (G97-A mAb)(bottom), respectively. Experiments were repeated 3 times with similar results. **b.** Flow cytometry analyses of GPR97-ligand$^+$ cells in different blood cell populations as indicated. Data shown are the results of one representative of at least 10 independent experiments with similar results. **c.** Human neutrophil-specific feature of the GPR97-ligand interaction. FACS-based ligand-binding assays were performed in purified human and mouse neutrophils using human and mouse GPR97^E-mFc probes as indicated. **d, e.** Morphological changes of neutrophils induced by GPR97^E-mFc were detected by flow cytometry analyses either shown as FSC/SSC plots (**d**) or FSC/F-actin staining (**e**). Photos within (**e**) were fluorescence images of cells stained with FITC-phalloidin. Scale bar, 20 µm. **f, g.** FACS analyses of ROS production (n = 5 independent experiments) (**f**) and expression of defined CD markers (CD16 n = 5, others n = 3 independent experiments) (**g**) in neutrophils treated as indicated. **h.** Time-dependent IL-8 production of neutrophils treated as indicated. (n = 3 independent experiments). Data in **f-h** are presented as means ± SEM. P value was determined by one-way ANOVA. Source data are provided in the Source Data file.

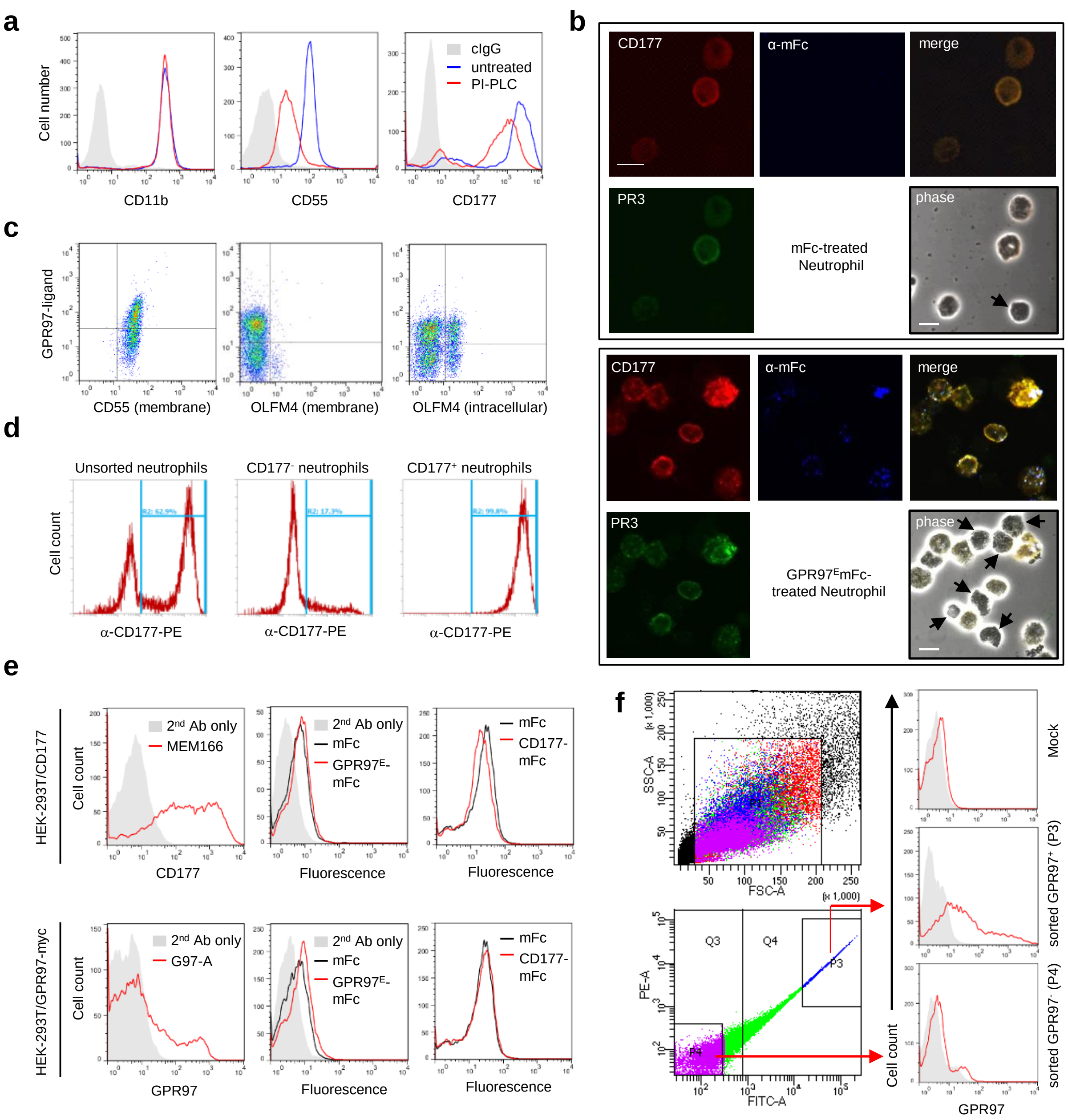

a
Cell number
CD11b
CD55
CD177
cIgG
untreated
PI-PLC
b
CD177
α-mFc
merge
PR3
mFc-treated Neutrophil
phase
GPR97EmFc-treated Neutrophil
c
GPR97-ligand
CD55 (membrane)
OLFM4 (membrane)
OLFM4 (intracellular)
d
Unsorted neutrophils
CD177- neutrophils
CD177+ neutrophils
Cell count
α-CD177-PE
e
HEK-293T/CD177
Cell count
2nd Ab only
MEM166
CD177
mFc
GPR97E-mFc
Fluorescence
CD177-mFc
HEK-293T/GPR97-myc
G97-A
GPR97
f
SSC-A
FSC-A
PE-A
FITC-A
Q3
Q4
P3
P4
Cell count
Mock
sorted GPR97+ (P3)
sorted GPR97- (P4)

Supplementary Fig. 2 Identification of mPR3 as the GPR97-specific binding partner on human neutrophils. **a.** Flow cytometry analyses of specific surface markers in neutrophils treated without or with phosphoinositide phospholipase C (PI-PLC). Markers included CD11b, CD55, and CD177. Both CD55 and CD177 are glycosylphosphatidylinositol-anchored proteins while CD11b is a transmembrane protein. **b.** Fluorescence staining of mPR3 (green) and CD177 (red) in neutrophils treated with mFc or GPR97^{E}-mFc. The mFc-fusion protein probes were detected using anti-mFc mAb (blue). Arrows indicated CD177^{-} neutrophils. Scale bar, 20 µm. Experiments were repeated 3 times with similar results. **c.** Dot-plots of flow cytometry analyses of neutrophils double-stained with GPR97^{E}-mFc and anti-CD55 or anti-OLFM4. neutrophils were fixed without or with membrane permeabilization to stain for membrane or intracellular proteins, respectively. **d.** Flow cytometry analyses of CD177 expression of neutrophils that were subjected to MACS sorting for the enrichment of CD177^{+} and CD177^{-} sub-populations. **e.** Ligand-binding assays showing no homotyic and heterotypic protein-protein interactions between GPR97 and CD177. HEK-293T transfectants expressing CD177 or GPR97 were incubated with GPR97^{E}-mFc or CD177-mFc proteins, washed, and stained with anti-mFc mAb followed by detection with the fluorescence-labeled 2nd Ab. GPR97 and CD177 expression in transfected cells were confirmed using anti-GPR97 (G97-A) and anti-CD177 (MEM166) mAbs, respectively. **f.** Flow cytometry plots showing the FACS-sorted GPR97^{high} and GPR97^{low} populations of HEK-293T transfectants. Source data are provided in the Source Data file.

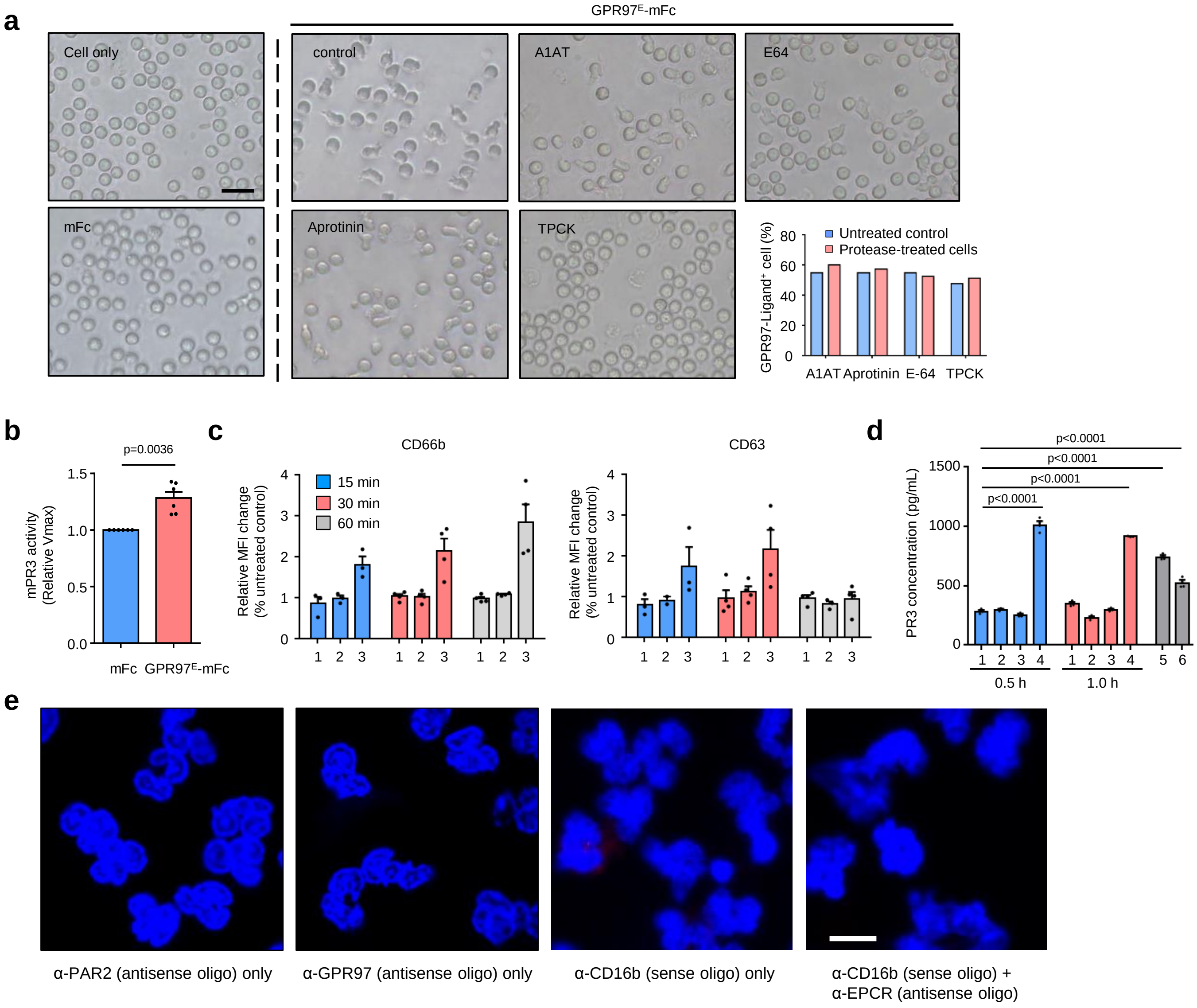
a
GPR97E-mFc
Cell only
control
A1AT
E64
mFc
Aprotinin
TPCK
GPR97-Ligand+ cell (%)
Untreated control
Protease-treated cells
A1AT Aprotinin E-64 TPCK
b
p=0.0036
mPR3 activity (Relative Vmax)
mFc GPR97E-mFc
c
CD66b
CD63
Relative MFI change (% untreated control)
15 min
30 min
60 min
d
p<0.0001
PR3 concentration (pg/mL)
0.5 h
1.0 h
e
α-PAR2 (antisense oligo) only
α-GPR97 (antisense oligo) only
α-CD16b (sense oligo) only
α-CD16b (sense oligo) + α-EPCR (antisense oligo)

Supplementary Fig. 3 GPR97 is a positive allosteric effector of neutrophil mPR3. **a.** Morphological changes of neutrophils treated with $GPR97^E$-mFc in the absence or presence of protease inhibitors as indicated. Cells only and mFc-treated cells were negative controls. Scale bar, 50 μm. The lower panel at right indicated similar percentages of GPR97-ligand$^+$ neutrophils in the absence or presence of protease inhibitors as indicated. Data shown are the results of one representative of 3 independent experiments with similar results. **b.** The relative Vmax of mPR3 enzymatic activity of neutrophils incubated with mFc and $GPR97^E$-mFc (n = 3). Data are presented as means ± SEM. P value was determined by two-sided unpaired student's t-test. **c, d.** Flow cytometry (**c**) and ELISA (**d**) analyses showing that $GPR97^E$-mFc treatment did not induce significant neutrophil degranulation. **c.** Relative cell surface CD66b and CD63 expression levels of neutrophils treated with mFc (# 1), $GPR97^E$-mFc (# 2), or fMLF (#3) for different time periods were compared with those of untreated cells (n = 3). CD66b and CD63 were used as degranulation markers of specific and azurophilc granules, respectively. **d.** Relative PR3 concentration changes detected by ELISA analysis of supernatants of neutrophils treated without (# 1) or with mFc (# 2), $GPR97^E$-mFc (# 3), or cytochalasin B + fMLF (# 4) for different time periods as indicated (n = 3). Cells treated with A23187 (1 μM) for 10 min (# 5) and 15 min (# 6) were included as positive controls. Data are presented as means ± SEM. P value was determined by one-way ANOVA. **e.** Results of the PLA analyses showing the minimal background signals when using mAb probes for single receptors and non-associated receptor pairs as indicated. Scale bar, 10 μm. Experiments were repeated 3 times with similar results. Source data are provided in the Source Data file.

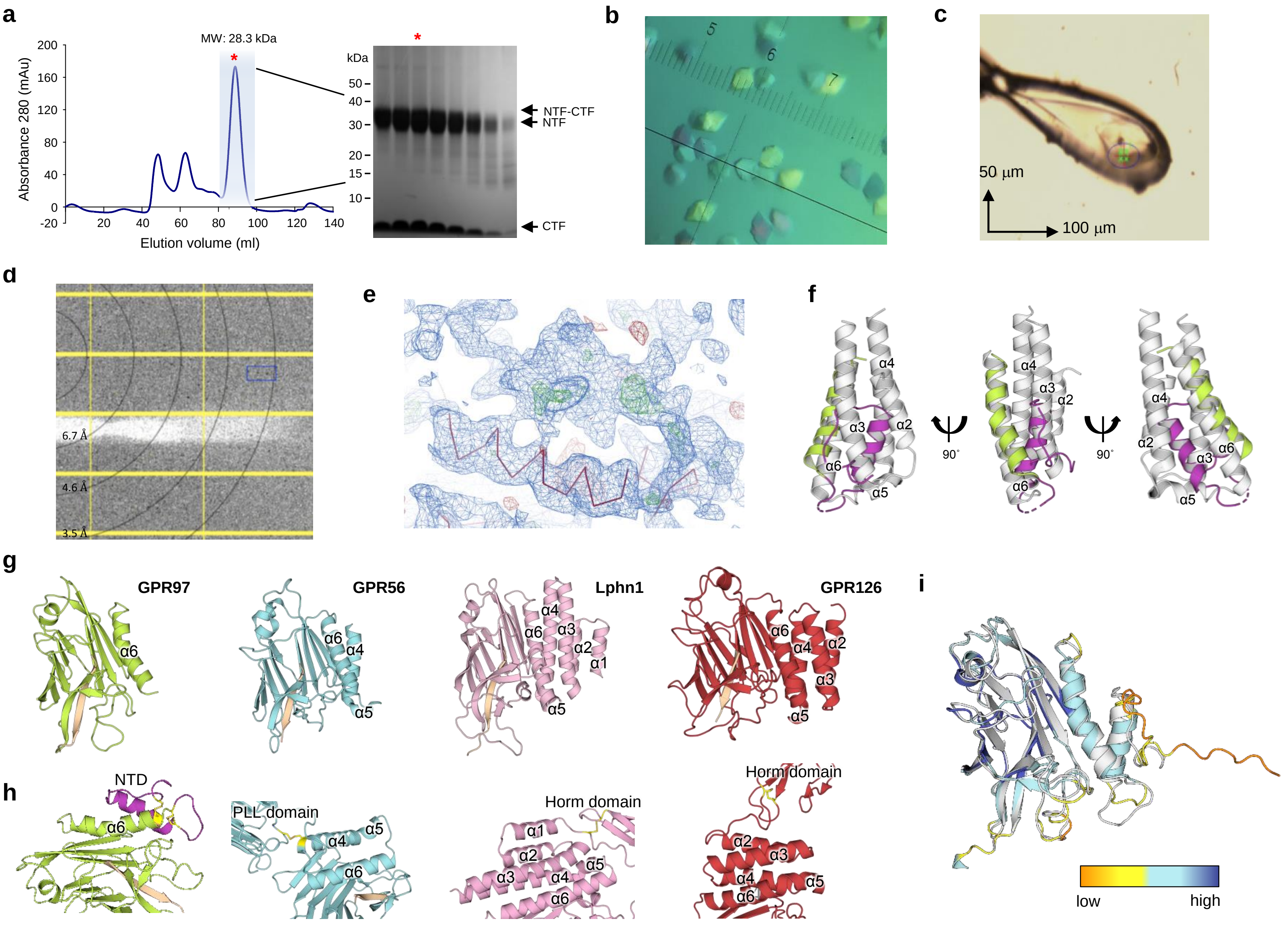
a
MW: 28.3 kDa
Absorbance 280 (mAu)
Elution volume (ml)
kDa
NTF-CTF
NTF
CTF
b
c
50 μm
100 μm
d
6.7 Å
4.6 Å
3.5 Å
e
f
α4
α3
α2
α6
α5
90˚
g
GPR97
GPR56
Lphn1
GPR126
h
NTD
PLL domain
Horm domain
i
low
high

Supplementary Fig. 4 Crystallographic studies of GPR97-ECR. **a.** Representative SEC elution profile and SDS-PAGE analysis of purified GPR97-ECR purification. The red asterisk indicated the GPR97-ECR-containing SEC peak and protein purity was assessed by coomassie blue staining in SDS-PAGE gels. GPR97-ECR run as two bands (NTF and CTF) on the SDS-PAGE gel due to partial autoproteolysis of its GAIN domain. **b.** GPR97-ECR crystals were grown in 1.8 M tri-ammonium citrate pH 7. **c.** Crystals were cryo-protected and individually assessed for diffraction quality. Experiments of **a-c** were repeated 3 times with similar results. **d.** A representative image from the native X-ray diffraction dataset collected at the ESRF beamline ID30B. Resolution rings are indicated. **e.** The α helix of GPR97 GAIN subdomain A is depicted in a red trace in a 2Fo-Fc electron density map shown at the 1 sigma level in blue. Clear additional density is seen on top of the GPR97 GAIN domain, accounting for the small NTD. The Fo-Fc electron density map is shown at +/- 3 sigma level in green and red, respectively. **f.** Structural alignment of the α helix from the GPR97 GAIN domain (green) with the α2-α6 helices from LPHN1 GAIN domain (white, PDB ID: 4DLQ) showing that the NTD (purple) is not part of the GAIN domain. **g.** Structural comparison of GPR97 GAIN domain with those crystallised and available in the PDB database (PDB IDs: 5KVM (GPR56), 4DLQ (Lphn1), 6V55 (GPR126)). **h.** Close-up of the interdomain disulphide bridges linking the GAIN domain of GPR97, GPR56, Lphn1 and GPR126 to the NTD, PLL domain, Horm domain and Horm domain, respectively. **i.** Structural comparison of the model predicted by alpha-fold with our experimental model (white). The alpha-fold model is coloured from very low model confidence (orange) to very high model confidence (blue). Source data are provided in the Source Data file.

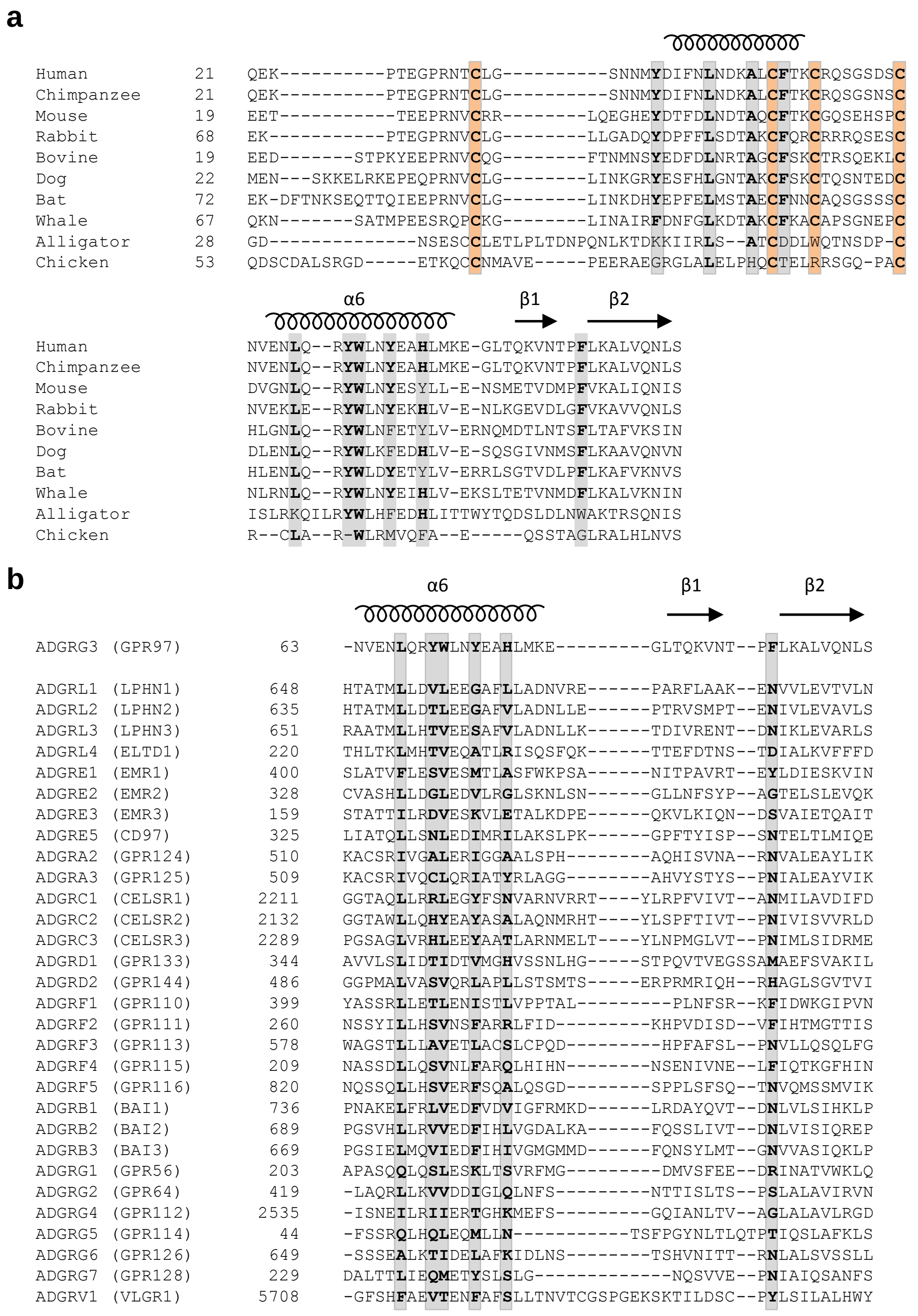
a
Human 21 QEK----------PTEGPRNTCLG----------SNNMYDIFNLNDKALCFTKCRQSGSDSC
Chimpanzee 21 QEK----------PTEGPRNTCLG----------SNNMYDIFNLNDKALCFTKCRQSGSNSC
Mouse 19 EET-----------TEEPRNVCRR--------LQEGHEYDTFDLNDTAQCFTKCGQSEHSPC
Rabbit 68 EK-----------PTEGPRNVCLG--------LLGADQYDPFFLSDTAKCFQRCRRRQSESC
Bovine 19 EED-------STPKYEEPRNVCQG--------FTNMNSYEDFDLNRTAGCFSKCTRSQEKLC
Dog 22 MEN---SKKELRKEPEQPRNVCLG--------LINKGRYESFHLGNTAKCFSKCTQSNTEDC
Bat 72 EK-DFTNKSEQTTQIEEPRNVCLG--------LINKDHYEPFELMSTAECFNNCAQSGSSSC
Whale 67 QKN-------SATMPEESRQPCKG--------LINAIRFDNFGLKDTAKCFKACAPSGNEPC
Alligator 28 GD-------------NSESCCLETLPLTDNPQNLKTDKKIIRLS--ATCDDLWQTNSDP-C
Chicken 53 QDSCDALSRGD-----ETKQCCNMAVE-----PEERAEGRGLALELPHQCTELRRSGQ-PAC
α6
β1
β2
Human NVENLQ--RYWLNYEAHLMKE-GLTQKVNTPFLKALVQNLS
Chimpanzee NVENLQ--RYWLNYEAHLMKE-GLTQKVNTPFLKALVQNLS
Mouse DVGNLQ--RYWLNYESYLL-E-NSMETVDMPFVKALIQNIS
Rabbit NVEKLE--RYWLNYEKHLV-E-NLKGEVDLGFVKAVVQNLS
Bovine HLGNLQ--RYWLNFETYLV-ERNQMDTLNTSFLTAFVKSIN
Dog DLENLQ--RYWLKFEDHLV-E-SQSGIVNMSFLKAAVQNVN
Bat HLENLQ--RYWLDYETYLV-ERRLSGTVDLPFLKAFVKNVS
Whale NLRNLQ--RYWLNYEIHLV-EKSLTETVNMDFLKALVKNIN
Alligator ISLRKQILRYWLHFEDHLITTWYTQDSLDLNWAKTRSQNIS
Chicken R--CLA--R-WLRMVQFA--E-----QSSTAGLRALHLNVS
b
α6
β1
β2
ADGRG3 (GPR97) 63 -NVENLQRYWLNYEAHLMKE---------GLTQKVNT--PFLKALVQNLS
ADGRL1 (LPHN1) 648 HTATMLLDVLEEGAFLLADNVRE------PARFLAAK--ENVVLEVTVLN
ADGRL2 (LPHN2) 635 HTATMLLDTLEEGAFVLADNLLE------PTRVSMPT--ENIVLEVAVLS
ADGRL3 (LPHN3) 651 RAATMLLHTVEESAFVLADNLLK------TDIVRENT--DNIKLEVARLS
ADGRL4 (ELTD1) 220 THLTKLMHTVEQATLRISQSFQK------TTEFDTNS--TDIALKVFFFD
ADGRE1 (EMR1) 400 SLATVFLESVESMTLASFWKPSA------NITPAVRT--EYLDIESKVIN
ADGRE2 (EMR2) 328 CVASHLLDGLEDVLRGLSKNLSN------GLLNFSYP--AGTELSLEVQK
ADGRE3 (EMR3) 159 STATTILRDVESKVLETALKDPE------QKVLKIQN--DSVAIETQAIT
ADGRE5 (CD97) 325 LIATQLLSNLEDIMRILAKSLPK------GPFTYISP--SNTELTLMIQE
ADGRA2 (GPR124) 510 KACSRIVGALERIGGAALSPH--------AQHISVNA--RNVALEAYLIK
ADGRA3 (GPR125) 509 KACSRIVQCLQRIATYRLAGG--------AHVYSTYS--PNIALEAYVIK
ADGRC1 (CELSR1) 2211 GGTAQLLRRLEGYFSNVARNVRRT----YLRPFVIVT--ANMILAVDIFD
ADGRC2 (CELSR2) 2132 GGTAWLLQHYEAYASALAQNMRHT----YLSPFTIVT--PNIVISVVRLD
ADGRC3 (CELSR3) 2289 PGSAGLVRHLEEYAATLARNMELT----YLNPMGLVT--PNIMLSIDRME
ADGRD1 (GPR133) 344 AVVLSLIDTIDTVMGHVSSNLHG-----STPQVTVEGSSAMAEFSVAKIL
ADGRD2 (GPR144) 486 GGPMALVASVQRLAPLLSTSMTS-----ERPRMRIQH--RHAGLSGVTVI
ADGRF1 (GPR110) 399 YASSRLLETLENISTLVPPTAL---------PLNFSR--KFIDWKGIPVN
ADGRF2 (GPR111) 260 NSSYILLHSVNSFARRLFID---------KHPVDISD--VFIHTMGTTIS
ADGRF3 (GPR113) 578 WAGSTLLLAVETLACSLCPQD---------HPFAFSL--PNVLLQSQLFG
ADGRF4 (GPR115) 209 NASSDLLQSVNLFARQLHIHN--------NSENIVNE--LFIQTKGFHIN
ADGRF5 (GPR116) 820 NQSSQLLHSVERFSQALQSGD--------SPPLSFSQ--TNVQMSSMVIK
ADGRB1 (BAI1) 736 PNAKELFRLVEDFVDVIGFRMKD------LRDAYQVT--DNLVLSIHKLP
ADGRB2 (BAI2) 689 PGSVHLLRVVEDFIHLVGDALKA------FQSSLIVT--DNLVISIQREP
ADGRB3 (BAI3) 669 PGSIELMQVIEDFIHIVGMGMMD------FQNSYLMT--GNVVASIQKLP
ADGRG1 (GPR56) 203 APASQQLQSLESKLTSVRFMG---------DMVSFEE--DRINATVWKLQ
ADGRG2 (GPR64) 419 -LAQRLLKVVDDIGLQLNFS---------NTTISLTS--PSLALAVIRVN
ADGRG4 (GPR112) 2535 -ISNEILRIIERTGHKMEFS---------GQIANLTV--AGLALAVLRGD
ADGRG5 (GPR114) 44 -FSSRQLHQLEQMLLN-----------TSFPGYNLTLQTPTIQSLAFKLS
ADGRG6 (GPR126) 649 -SSSEALKTIDELAFKIDLNS--------TSHVNITT--RNLALSVSSLL
ADGRG7 (GPR128) 229 DALTTLIEQMETYSLSLG-------------NQSVVE--PNIAIQSANFS
ADGRV1 (VLGR1) 5708 -GFSHFAEVTENFAFSLLTNVTCGSPGEKSKTILDSC--PYLSILALHWY

Supplementary Fig. 5 The NTD is stabilised by hydrophobic interactions conserved in all GPR97 orthologs, but not found in other GAIN domains. **a.** Sequence alignment with secondary structure of the NTD, α6 helix and β1-β2 strands of the GAIN domain of GPR97 orthologs. The conserved cysteines involved in known disulphides bridges are shown in bold and highlighted in yellow. The conserved residues involved in the hydrophobic interactions stabilising the NTD to the GAIN domain are shown in bold and highlighted in grey. **b.** Sequence alignment with secondary structure of the α6 helix and β1-β2 strands of the GAIN domain of all aGPCRs. The residues involved in stabilising GPR97 NTD to its GAIN domain are shown in bold and highlighted in grey. Source data are provided in the Source Data file.

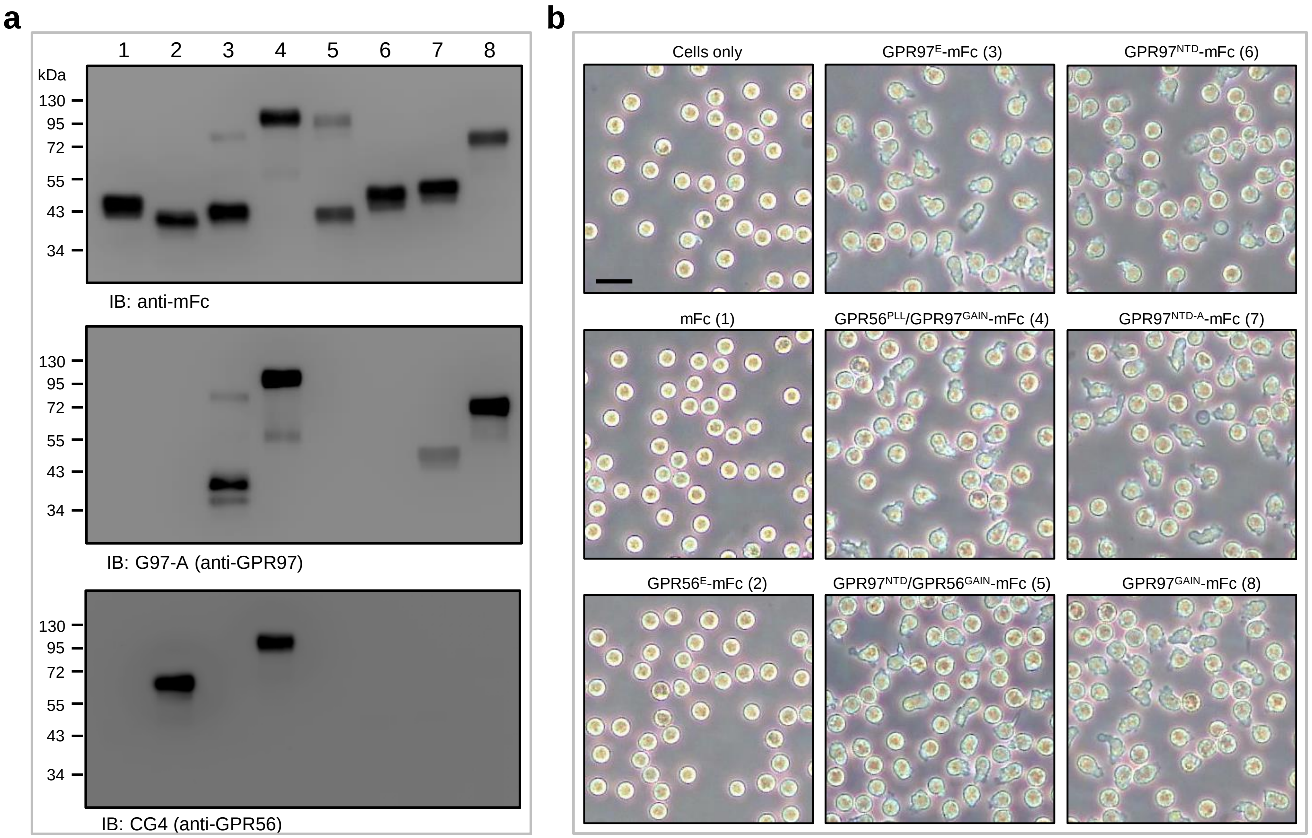
a
1 2 3 4 5 6 7 8
kDa
130
95
72
55
43
34
IB: anti-mFc
IB: G97-A (anti-GPR97)
IB: CG4 (anti-GPR56)
b
Cells only
GPR97E-mFc (3)
GPR97NTD-mFc (6)
mFc (1)
GPR56PLL/GPR97GAIN-mFc (4)
GPR97NTD-A-mFc (7)
GPR56E-mFc (2)
GPR97NTD/GPR56GAIN-mFc (5)
GPR97GAIN-mFc (8)

Supplementary Fig. 6 Mapping of the mPR3-binding regions in GPR97-ECR. **a.** Western blot analyses of the domain-swapped and domain-truncated $GPR97^{E}$-mFc probes. Lane 1, mFc only; 2, $GPR56^{E}$-mFc; 3, $GPR97^{E}$-mFc; 4, $GPR56^{PLL}$/$GPR97^{GAIN}$-mFc; 5, $GPR97^{NTD}$/$GPR56^{GAIN}$-mFc; 6, $GPR97^{NTD}$-mFc; 7, $GPR97^{NTD\text{-}A}$-mFc; 8, $GPR97^{GAIN}$-mFc. Blots were probed with anti-mFc (top panel), G97-A (anti-GPR97 mAb)(middle panel), and CG4 (anti-GPR56 mAb)(lower panel), respectively. **b.** Morphological changes of neutrophils induced by various $GPR97^{E}$-mFc probes as indicated. Cells only and those treated with mFc and $GPR56^{E}$-mFc were included as negative controls. Scale bar, 50 µm. Experiments were repeated 3 times with similar results. Source data are provided in the Source Data file.

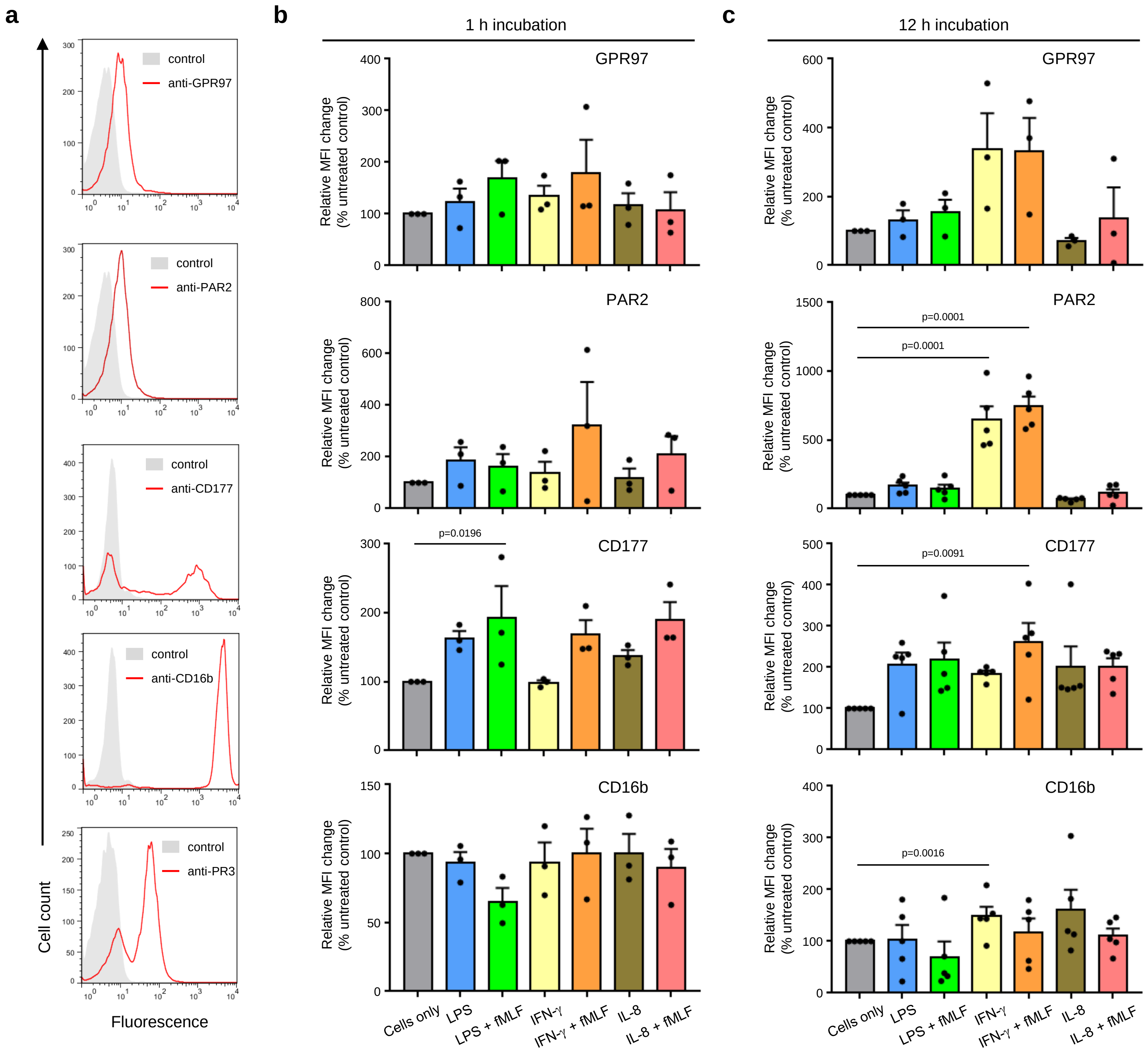
a
b
c
1 h incubation
12 h incubation
Cell count
Fluorescence
control
anti-GPR97
anti-PAR2
anti-CD177
anti-CD16b
anti-PR3
Relative MFI change (% untreated control)
GPR97
PAR2
CD177
CD16b
p=0.0196
p=0.0001
p=0.0001
p=0.0091
p=0.0016
Cells only
LPS
LPS + fMLF
IFN-γ
IFN-γ + fMLF
IL-8
IL-8 + fMLF

Supplementary Fig. 7 Expressional regulation of GPR97 and PAR2 in neutrophils. **a.** Representative expression profiles of indicated receptors in resting human neutrophils detected by flow cytometry. **b, c.** Flow cytometry analyses of GPR97, PAR2, CD177, and CD16b expression in resting neutrophils treated without or with various inflammatory stimuli (LPS, 200 ng/mL; IFN-γ, 200 U/mL; IL-8, 400 ng/mL; fMLF, 1 μM) for 1 h (n = 3) (**b**) or 12 h (n = 5, except GPR97 n = 3) (**c**). Data are presented as means ± SEM. P value was determined by one-way ANOVA. Source data are provided in the Source Data file.

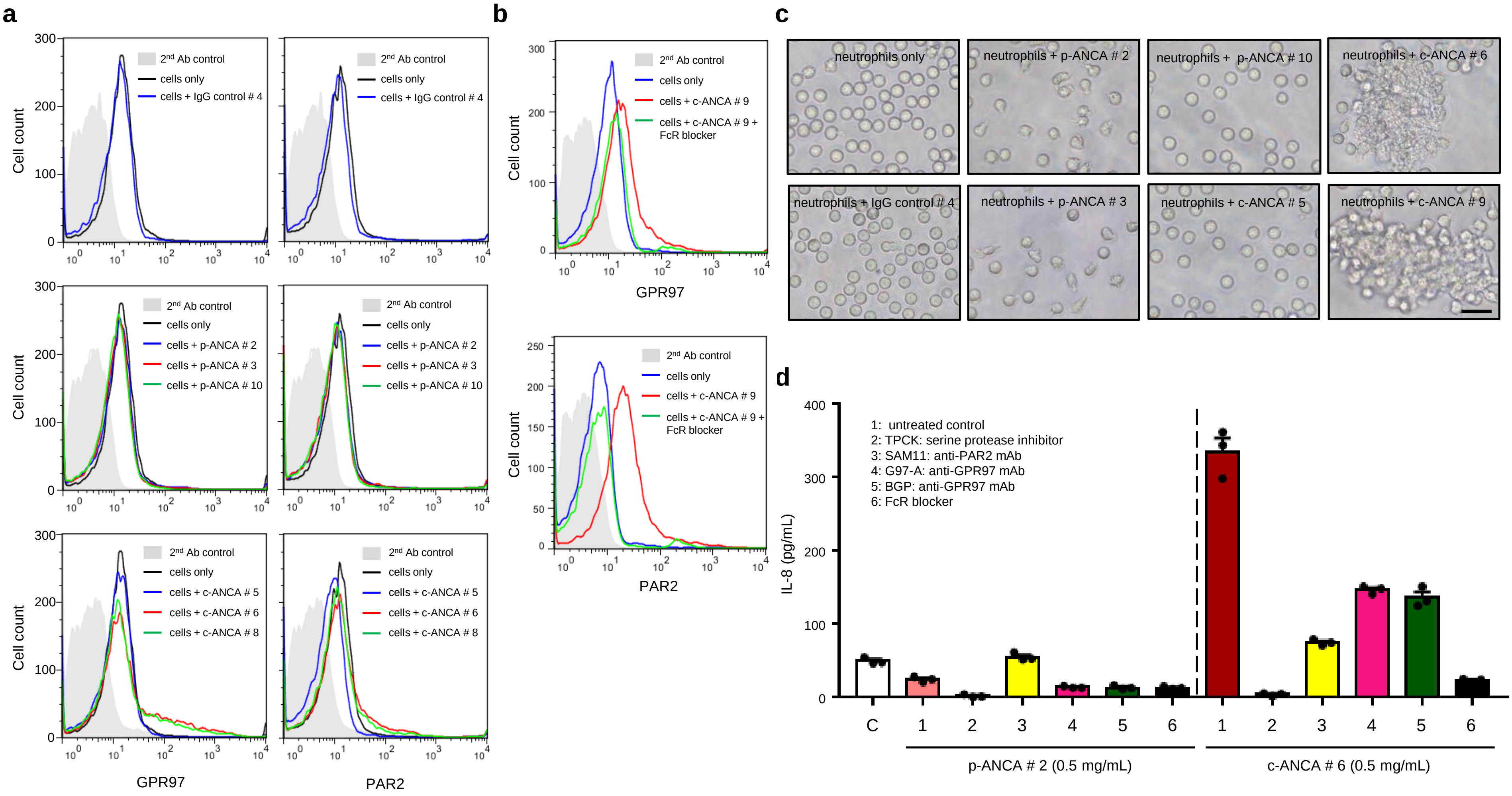
a
b
c
d
Cell count
GPR97
PAR2
2nd Ab control
cells only
cells + IgG control # 4
cells + p-ANCA # 2
cells + p-ANCA # 3
cells + p-ANCA # 10
cells + c-ANCA # 5
cells + c-ANCA # 6
cells + c-ANCA # 8
cells + c-ANCA # 9
cells + c-ANCA # 9 + FcR blocker
neutrophils only
neutrophils + p-ANCA # 2
neutrophils + p-ANCA # 10
neutrophils + c-ANCA # 6
neutrophils + IgG control # 4
neutrophils + p-ANCA # 3
neutrophils + c-ANCA # 5
neutrophils + c-ANCA # 9
IL-8 (pg/mL)
1: untreated control
2: TPCK: serine protease inhibitor
3: SAM11: anti-PAR2 mAb
4: G97-A: anti-GPR97 mAb
5: BGP: anti-GPR97 mAb
6: FcR blocker
C
p-ANCA # 2 (0.5 mg/mL)
c-ANCA # 6 (0.5 mg/mL)

Supplementary Fig. 8 FcR-dependent up-regulated expression of GPR97 and PAR2 in neutrophils. **a.** Flow cytometry analysis of up-regulated GPR97 and PAR2 expression in neutrophils incubated with purified PR3-ANCA IgGs but not purified MPO-ANCA IgGs as indicated. **b.** Representative flow cytometry analyses of FcR-dependent GPR97 and PAR2 up-regulated expression in neutrophils incubated with purified PR3-ANCA IgGs. **c.** Representative light microscope images showing aggregates of neutrophils incubated with purified PR3-ANCA IgGs but not purified MPO-ANCA IgGs as indicated. Neutrophils only and those treated with control IgGs from a normal subject were included as negative control. Scale bar, 50 µm. **d.** ELISA analyses of IL-8 secreted by neutrophils incubated with purified PR3-ANCA or MPO-ANCA IgGs in the absence or presence of protease inhibitors or blocking reagents as indicated. Data are presented as means ± SEM of one representative experiment done in triplicate. Source data are provided in the Source Data file.

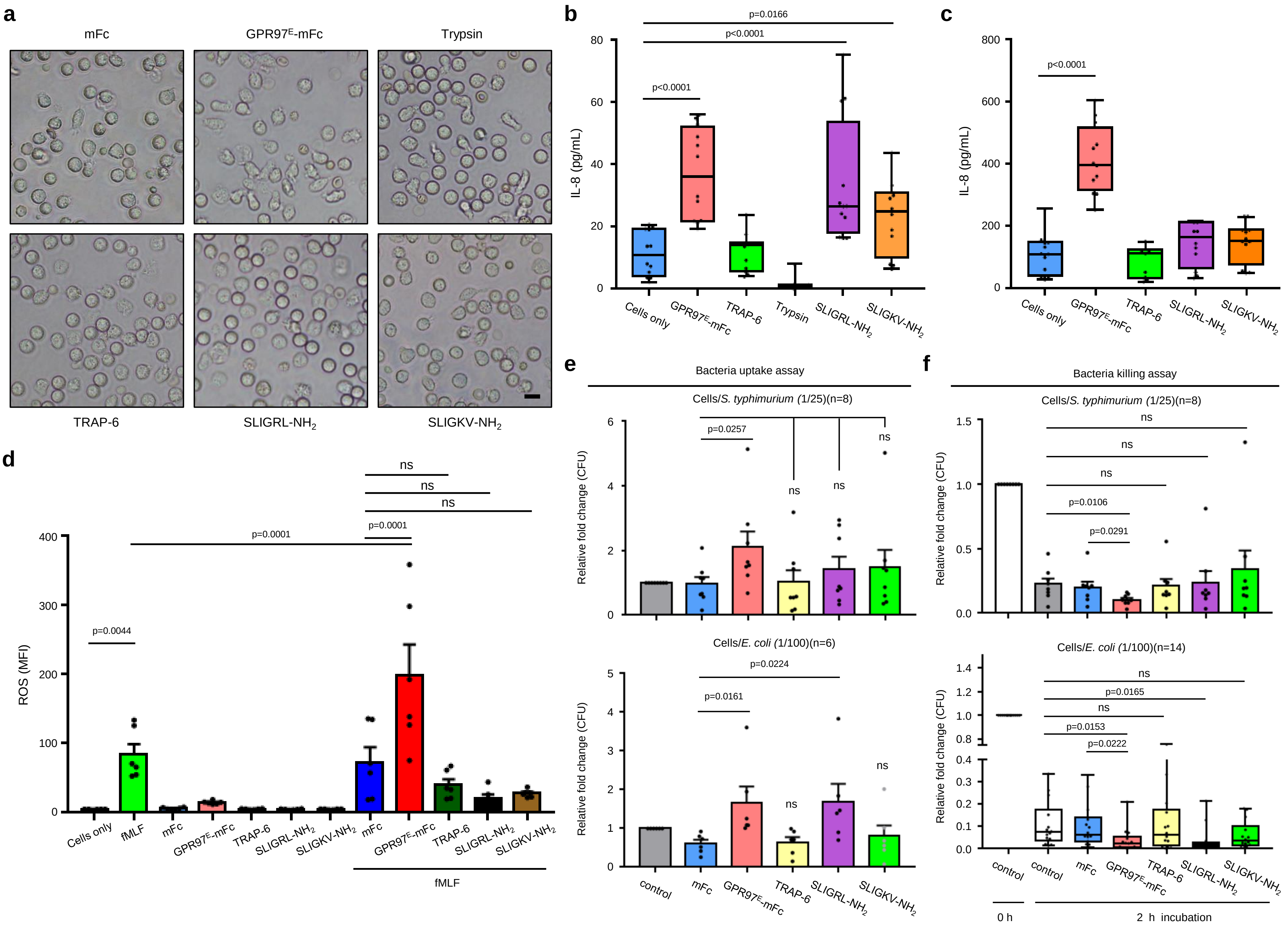
a
mFc
GPR97E-mFc
Trypsin
TRAP-6
SLIGRL-NH2
SLIGKV-NH2
b
IL-8 (pg/mL)
p=0.0166
p<0.0001
p<0.0001
Cells only
GPR97E-mFc
TRAP-6
Trypsin
SLIGRL-NH2
SLIGKV-NH2
c
IL-8 (pg/mL)
p<0.0001
Cells only
GPR97E-mFc
TRAP-6
SLIGRL-NH2
SLIGKV-NH2
d
ROS (MFI)
p=0.0044
p=0.0001
p=0.0001
ns
ns
ns
Cells only
fMLF
mFc
GPR97E-mFc
TRAP-6
SLIGRL-NH2
SLIGKV-NH2
mFc
GPR97E-mFc
TRAP-6
SLIGRL-NH2
SLIGKV-NH2
fMLF
e
Bacteria uptake assay
Cells/S. typhimurium (1/25)(n=8)
Relative fold change (CFU)
p=0.0257
ns
ns
ns
Cells/E. coli (1/100)(n=6)
p=0.0224
p=0.0161
ns
ns
control
mFc
GPR97E-mFc
TRAP-6
SLIGRL-NH2
SLIGKV-NH2
f
Bacteria killing assay
Cells/S. typhimurium (1/25)(n=8)
Relative fold change (CFU)
ns
ns
ns
p=0.0106
p=0.0291
Cells/E. coli (1/100)(n=14)
ns
p=0.0165
ns
p=0.0153
p=0.0222
control
control
mFc
GPR97E-mFc
TRAP-6
SLIGRL-NH2
SLIGKV-NH2
0 h
2 h incubation

Supplementary Figure 9: Comparison of neutrophil activation phenotypes triggered by PAR2-specific agonists and GPR97^{E}-mFc. **a.** Morphologies of neutrophils treated without or with GPR97^{E}-mFc (10 μg/mL) or various PAR2 activation triggers as indicated (trypsin, 1 μg/mL; SLIGRL-NH_2 agonistic peptide, 100 μM; SLIGKV-NH_2 agonistic peptide, 100 μM) for 1 h. The PAR1-specific TRAP-6 agonistic peptide (30 μM) was included as a negative control. Scale bar, 20 μm. Experiments were repeated 3 times with similar results. **b, c.** ELISA analysis of IL-8 secreted by neutrophils treated as indicated in serum-free medium (**b**) or RPMI complete medium (**c**) for 3 h. n = 4 independent experiments. Data are presented as means ± SEM. P value was determined by one-way ANOVA. Note that while comparable IL-8 levels were up-regulated in neutrophils incubated with GPR97^{E}-mFc and PAR2-agonistic peptides in serum-free medium (**b**), its concentrations were of very low levels compared to those secreted from cells cultured in RPMI complete medium in which only GPR97^{E}-mFc, but not PAR2-agonistic peptides, induced significantly increased IL-8 (**c**). **d.** Analysis of ROS production by neutrophils treated without or with various triggers in the absence or presence of fMLF (1 μM) as indicated. mFc was included as a negative control. n = 3 independent experiments. Data are presented as means ± SEM. P value was determined by one-way ANOVA. **e, f.** Phagocytosis (**e**) and killing (**f**) of live bacteria (*E. coli*, n= 6 for phagocytosis assay n = 14 for killing assay, and *S. typhimurium* n= 8 for both assays) by neutrophils incubated without or with GPR97^{E}-mFc and PAR2-agonistic peptides as indicated. mFc and TRAP-6 were included as negative controls. Data are presented as means ± SEM. P value was determined by one-way ANOVA. ns, non-significant. Source data are provided in the Source Data file.

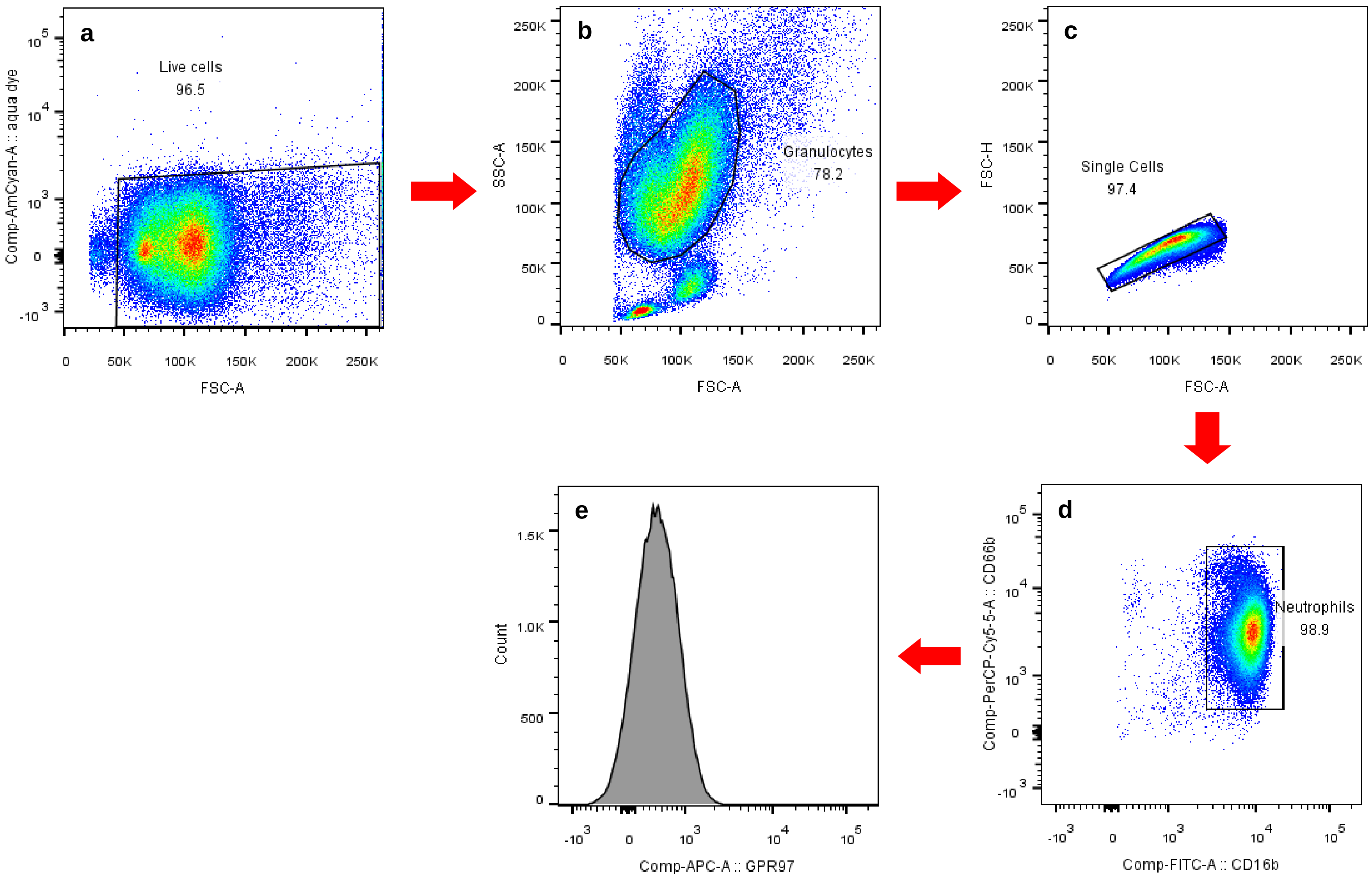
a
Live cells
96.5
Comp-AmCyan-A :: aqua dye
FSC-A
b
Granulocytes
78.2
SSC-A
FSC-A
c
Single Cells
97.4
FSC-H
FSC-A
d
Neutrophils
98.9
Comp-PerCP-Cy5-5-A :: CD66b
Comp-FITC-A :: CD16b
e
Count
Comp-APC-A :: GPR97

Supplementary Figure 10: FACS strategy of gating human neutrophil leukocytes from blood samples for FACS data in the Figure 1**d**. Viable blood cells were gated negatively on aqua fluorescent dye (**a**) and then granulocytes were gated by FSC versus SSC graph (**b**). Subsequently, single cells (**c**) from total granulocytes were gated for CD16b$^+$ neutrophils (**d**) and the mean fluorescent intensity of GPR97 on this CD16b$^+$ population was determined (**e**).

Supplementary Table 1 Antibody list

Antibody	Antibody #Catalog	Clone	Assay
anti-CD11b	BD Biosciences #555388	ICRF44	FACS (5 μg/mL)
anti-CD62L	BD Biosciences #555544	DREG-56	FACS (5 μg/mL)
anti-CD11a	BD Biosciences #555379	G43-25B	FACS (5 μg/mL)
anti-CD16	BD Biosciences #555407	3G8	FACS (5 μg/mL)
anti-CD44	BD Biosciences #555478	G44-26	FACS (5 μg/mL)
anti-CD54	BD Biosciences #559771	HA58	FACS (5 μg/mL)
anti-CXCR1	BD Biosciences #555939	5A12	FACS (5 μg/mL)
anti-CXCR4	BD Biosciences # 555974	12G5	FACS (5 μg/mL)
anti-CD51	BD Biosciences #565835	NKI-M9	FACS (5 μg/mL)
anti-CD18	BD Biosciences #555924	6.7	FACS (5μg/ml)
anti-CD18	BioLegend #302108	TS1/18	FACS (5 μg/mL)
anti-CD41/CD61	BioLegend #359810	A2A9/6	FACS (5 μg/mL)
anti-CD62P	BioLegend #304906	AK4	FACS (5 μg/mL)
anti-CD63	BD Biosciences #557288	H5C6	FACS (5 μg/mL)
anti-CD66b	BD Biosciences #561645	G10F5	FACS (5 μg/mL)
anti-CD177	BioLegend #315802	MEM-166	Functional assay (5 μg/mL)
anti-CD177	BioLegend #315806	MEM-166	FACS, Confocol (5 μg/mL)
anti-CD177	Santa Cruz sc-376329	C5	Western blot (1:500), PLA, Functional assay (5 μg/mL)
anti-Thrombin R	Santa Cruz sc-13503	ATAP2	FACS (5 μg/mL)
anti-CD32	Gene Tex GTX-14572	IV.3	Functional assay (5 μg/mL)
anti-PR3	Thermo Fisher MA-11945	MCPR3-2	Western blot, FACS, Confocol, Functional assay, ELISA (5 μg/mL)
anti-PR3	Sanquin	CLB-12.8	FACS, Confocol, Functional assay (5 μg/mL)
anti-PR3	EURO DIAGNOSTICA	4A5	Functional assay (2 μL/test)
anti-OLFM4	abcam ab85046	Polyclonal	FACS (5 μg/mL)
anti-PAR2	Santa Cruz sc-13504	SAM11	Western blot, FACS, PLA, Functional assay (5 μg/mL)
anti-PAR2	R&D MAB3949	#344222	Functional assay (5 μg/mL)
anti-PAR2	R&D FAB3949P	#344222	FACS (5 μg/mL)
normal mouse IgG_1-APC	Santa Cruz sc-2888		FACS (5 μg/mL)
normal mouse IgG_1-PE	Santa Cruz sc-2866		FACS (5 μg/mL)
FITC-conjugated AffiniPure Goat Anti-Rabbit IgG(H+L)	Jackson ImmunnoResearch #111-095-144	Polyclonal	FACS, Confocol (1:200)
Alexa Fluor 647-conjugated goat anti-mouse IgG	Thermo Fisher A-21235	Polyclonal	FACS, Confocol (1:400)
anti-EPCR	Santa Cruz sc-53982	RCR-49	FACS (5 μg/mL)
anti-CD55	Santa Cruz sc-59092	BRIC 216	Western blot, FACS, Functional assay (5 μg/mL)
anti-CD16	BioLegend #302002	3G8	Western blot, PLA (5 μg/mL)
anti-CD62E	BD Biosciences #551144	68-5H11	FACS (5 μg/mL)
anti-VCAM-1	BD Biosciences #561679	51-10C9	FACS (5 μg/mL)
anti-EMR2	Invitrogen #MA5-16474	2A1	FACS, Functional assay (5 μg/mL)
anti-GPR97	in house	G97-A	FACS, Western blot, PLA, Confocol, Functional assay (5 μg/mL)
anti-GPR97	in house	BGP	Functional assay (5 μg/mL)

Supplementary Table 2 Cell line list

Cell line	Source	Catalogue no.
HT1080	ATCC*	CCL-121™
NIH/3T3	ATCC	CRL-1658™
HeLa	ATCC	CCL-2™
M059K	ATCC	CRL-2365™
U-87 MG	ATCC	HTB-14™
C32	ATCC	CRL-1585™
COS-7	ATCC	CRL-1651™
MCF-7	ATCC	CRL-3435™
HEK-293T	ATCC	CRL-3216™
K-562	ATCC	CCL-243™
G5T/VGH	BCRC*	60194
A2058	ATCC	CRL-11147™
A375	ATCC	CRL-1619™
Jurkat	ATCC	TIB-152™
CHO-K1	ATCC	CCL-61™
MeWo	ATCC	HTB-65™
MEL-14	ATCC	HB-132™
THP-1	ATCC	TIB-202™
U-937	ATCC	CRL-1593.2™
Chang Liver	ATCC	CCL-13™
Hep-3B	ATCC	HB-8064™
Hep-G2	ATCC	HB-8065™
SK-Hep	ATCC	HTB-52™
SK-MEL5	ATCC	HTB-70™
RPMI-7951	ATCC	HTB-66™
HL-60	ATCC	CCL-240™
Raw264.7	ATCC	TIB-71™
HUVEC	BCRC	H-UV001

*ATCC: American Type Culture Collection

*BCRC: Bioresource Collection and Research Center

Supplementary Table 3 Primer list

**mFc-fusion protein**	**Primer**	**Sequence (5' → 3')**	**Enzyme**
GPR56^{E}-mFc	Forward	AATAAGCTTACAGGTGGTGACTTCCAAGAGT	HinDIII
	Reverse	AAGCGGCCGCCACCTCCACCGAGGAGACCA	NotI
CD177-mFc	Forward	AGTCCAGTGTGGTGGAATTC	EcoRI
	Reverse	AAAGCGGCCGCCTGAGAGGCAGGAGGCTGC	NotI
GPR97^{E}-mFc	Forward	ATGAATTCTTAAGCTTGGTACTCAATTCGAAGG	EcoRI
	Reverse	TAGCGGCCGCGCGTGTGAGGATATGCACCGT	NotI
GPR56^{PLL}/GPR97^{GAIN}-mFc	Forward (GPR56)	T7 primer: TAATACGACTCACTATAGGG	
	Reverse (GPR56)	GTCCACCGAGGCATTGTGAG	
	Forward (GPR97)	CACACGGCCGCTCACAATGCCTCGGTGGACAATGTGGAAAACTTGCAGAG	
	Reverse (GPR97)	CATTTGTGACACTCCTTGCAT for mFc sequence	
GPR97^{NTD}/GPR56^{GAIN}-mFc	Forward (GPR97)	T7 primer: TAATACGACTCACTATAGGG	
	Reverse (GPR97)	GTGAGCGGCCGTGTGGGGAGGACTGTGGAAGCAGGAGTCGCTGCCCGACT	
	Forward (GPR56)	TTCCACAGTCCTCCCCACAC	
	Reverse (GPR56)	CATTTGTGACACTCCTTGCAT for mFc sequence	
GPR97^{NTD}-mFc	Forward	T7 primer: TAATACGACTCACTATAGGG	
	Reverse	AAGCGGCCGCATTGCAGGAGTCGCTGCCCG	NotI
GPR97^{NTD-A}-mFc	Forward	T7 primer: TAATACGACTCACTATAGGG	
	R-primer	AAGCGGCCGCTTCCTTCATCAGATGGGCCT	NotI
GPR97^{GAIN}-mFc	Forward	AATGTGGAAAACTTGCAGAGATACTGGCTAAACTACGAGG	
	R-primer	CTCTGCAAGTTTTCCACATTCTGACCTGAGGTCGGGAGCA	

Supplementary Table 4 Crystallographic statistics of GPR97-ECR

DATA COLLECTION		
	Native	S-SAD
Source	ID30B (ESRF)	I23 (DLS)
Wavelength λ (Å)	0.97625	2.7552
No. of crystals	1	1
Resolution (Å)	96.28-3.37 (3.64-3.37)	172.68-3.50 (3.83-3.50)
Space group	$P\ 4_3\ 2_1\ 2$	$P\ 4_3\ 2_1\ 2$
Cell dimensions a, b, c (Å); α, β, γ (˚)	96.3, 96.3, 172.4 90, 90, 90	95.9, 95.9, 172.7 90, 90, 90
Unique reflections	12030 (2398)	10793 (2519)
Multiplicity	11.6 (12.2)	22.8 (21.0)
Completeness (%)	99.7 (99.4)	100 (100)
Wilson B ($Å^2$)	103.06	140.66
R_{merge} (%)	27.3 (123.8)	22.5 (359.3)
R_{meas} (%)	28.6 (129.2)	23.0 (368.3)
R_{pim} (%)	8.3 (36.4)	4.8 (79.8)
$CC_{1/2}$ (%)	99.6 (52.1)	99.9 (61.6)
Average I/σ(I)	6.5 (1.9)	9.9 (0.9)

Values in parenthesis are for the highest resolution shell

REFINEMENT	
Resolution (Å)	84.11-3.37
Reflections (Work / Free set)	10967 / 1040
R_{work} / R_{free} (%)	26.59 / 31.30
No. of atoms (protein)	3640
Mean B value (Å2) (protein)	134.5
R.m.s.d. bonds (Å)	0.005
R.m.s.d. angles (˚)	1.229
Ramachandran	
Favoured (%)	94.3
Allowed (%)	5.7
Outliers (%)	0.00
Molprobity score / percentile	2.03

R_{merge}: merging R-factor
R_{meas}: multiplicity-corrected merging R-factor
R_{pim}: precision-indicating merging R-factor
$CC_{1/2}$: correlation coefficients between random half data sets
R.m.s.d.: root mean square deviation from ideal geometry
