# Supplementary material for: GPR97 triggers inflammatory processes in human neutrophils via a macromolecular complex upstream of PAR2 activation
Source: Nat Commun. 2022 Oct 27;13:6385. doi: 10.1038/s41467-022-34083-1 (PMC9613636; doi:10.1038/s41467-022-34083-1)
Supplement: Supplementary file 3 — Reporting Summary [file 41467_2022_34083_MOESM3_ESM.pdf]

nature portfolio

Corresponding author(s): DBPR (NCOMMS-22-05955A)

Last updated by author(s): Sep 24, 2022

Reporting Summary

Nature Portfolio wishes to improve the reproducibility of the work that we publish. This form provides structure for consistency and transparency in reporting. For further information on Nature Portfolio policies, see our Editorial Policies and the Editorial Policy Checklist.

Statistics

For all statistical analyses, confirm that the following items are present in the figure legend, table legend, main text, or Methods section.

n/a	Confirmed	
☐	☒	The exact sample size (*n*) for each experimental group/condition, given as a discrete number and unit of measurement
☐	☒	A statement on whether measurements were taken from distinct samples or whether the same sample was measured repeatedly
☐	☒	The statistical test(s) used AND whether they are one- or two-sided<br>*Only common tests should be described solely by name; describe more complex techniques in the Methods section.*
☒	☐	A description of all covariates tested
☐	☒	A description of any assumptions or corrections, such as tests of normality and adjustment for multiple comparisons
☐	☒	A full description of the statistical parameters including central tendency (e.g. means) or other basic estimates (e.g. regression coefficient) AND variation (e.g. standard deviation) or associated estimates of uncertainty (e.g. confidence intervals)
☐	☒	For null hypothesis testing, the test statistic (e.g. *F*, *t*, *r*) with confidence intervals, effect sizes, degrees of freedom and *P* value noted<br>*Give P values as exact values whenever suitable.*
☒	☐	For Bayesian analysis, information on the choice of priors and Markov chain Monte Carlo settings
☒	☐	For hierarchical and complex designs, identification of the appropriate level for tests and full reporting of outcomes
☒	☐	Estimates of effect sizes (e.g. Cohen's *d*, Pearson's *r*), indicating how they were calculated

Our web collection on statistics for biologists contains articles on many of the points above.

Software and code

Policy information about availability of computer code

Data collection	BD CellQuest Pro Version 6.0; ZEISS LSM780 ZEN 2011; Olympus FluoView FV10i, PyMOL v.2.3.4.
Data analysis	FlowJo v7.6.1 software; ZEISS ZEN 2011 software; Olympus FluoView FV10i software; Graphpad prism v6 and v7, DIALS 1.9.3, Coot 0.9.6, Aimless 0.7.3, Phaser 2.8.3, Parrot 1.0.4, CCP4 suite 7.1, ANODE 2013/1, autoBUSTER 2.10.4, autoPROC 1.0.5, STARANISO V3.347

For manuscripts utilizing custom algorithms or software that are central to the research but not yet described in published literature, software must be made available to editors and reviewers. We strongly encourage code deposition in a community repository (e.g. GitHub). See the Nature Portfolio guidelines for submitting code & software for further information.

Data

Policy information about availability of data

All manuscripts must include a data availability statement. This statement should provide the following information, where applicable:

- Accession codes, unique identifiers, or web links for publicly available datasets
- A description of any restrictions on data availability
- For clinical datasets or third party data, please ensure that the statement adheres to our policy

All data generated in this study are available in this manuscript, supplementary information, and Source Data files. A data availability statement is included in the manuscript. Information on antibodies used is provided in the antibody list in Supplementary Table 1 and in the Methods section. Uncropped gels are provided in the Source Data file. The immunoblotting data generated in this study are provided in the Source Data file. The statistical raw data are provided in the Source data file. Source data are provided with this paper. The X-ray structure data of GPR97 extracellular domain generated in this study have been deposited in the database of Protein Data Bank in Europe (PDBe) under accession code PDB ID 7QU8 (Deposition ID: D_1292120102) (https://doi.org/10.2210/pdb7QU8/pdb)

Field-specific reporting

Please select the one below that is the best fit for your research. If you are not sure, read the appropriate sections before making your selection.

☒ Life sciences ☐ Behavioural & social sciences ☐ Ecological, evolutionary & environmental sciences

For a reference copy of the document with all sections, see nature.com/documents/nr-reporting-summary-flat.pdf

Life sciences study design

All studies must disclose on these points even when the disclosure is negative.

Sample size	The minimal sample size needed to produce reliable measurements was determined for each type of analysis individually based on existing literature and our previous experience (e.g. PMID: 30559745, PMID: 28240246, or PMID: 31928845). Sample size was determined as being adequate by the magnitude and consistency of measurable differences between experimental groups. At least 3 independent experiments done in triplicate was considered significant for statistical analysis.
Data exclusions	No outliers were excluded.
Replication	The reproducibility of experiments was performed using cells isolated from different donors to confirm the experimental findings. Number of each experiment is given in the manuscript. All experiments were performed in triplicates.
Randomization	Sample randomization was not relevant to this study as each sample was divided into negative-, positive-control, and experimental groups and tested simultaneously in various experimental settings.
Blinding	Evaluation of the IHC results were done in blind by two different pathologists. Analysis of neutrophils from healthy control and patients was done in blind. Blinding samples of different cell types and primary cells were not relevant to this study as there is no prior knowledge of the expression characteristics of GPR97-ligand.

Reporting for specific materials, systems and methods

We require information from authors about some types of materials, experimental systems and methods used in many studies. Here, indicate whether each material, system or method listed is relevant to your study. If you are not sure if a list item applies to your research, read the appropriate section before selecting a response.

Materials & experimental systems

n/a	Involved in the study	
☐	☒	Antibodies
☐	☒	Eukaryotic cell lines
☒	☐	Palaeontology and archaeology
☒	☐	Animals and other organisms
☐	☒	Human research participants
☒	☐	Clinical data
☒	☐	Dual use research of concern

Methods

n/a	Involved in the study	
☒	☐	ChIP-seq
☐	☒	Flow cytometry
☒	☐	MRI-based neuroimaging

Antibodies

Antibodies used

The information of antibody used in this study, including the supplier's name, catalog number, and clone name as well as the dilution or concentration used are listed in the Supplementary Table 1 and in the Reporting Summary.
Anti-human CD11a, clone G43-25B; cat. no. 555379 (BD Biosciences, USA) (5 µg/mL); application, FACS analysis.
Anti-human CD11b, clone ICRF44; cat. no. 555388 (BD Biosciences, USA) (5 µg/mL); application, FACS analysis.
Anti-human CD16, clone 3G8; cat. no. 555407 (BD Biosciences, USA) (5 µg/mL); application, FACS analysis.
Anti-human CD16, clone 3G8; cat. no. 302002 (BioLegend, USA) (5 µg/mL); application, FACS and PLA analysis.
Anti-human CD16b-FITC , clone REA589; cat. no. 130-126-529 (Miltenyi Biotec) (2 µL/test); application, FACS sorting.
Anti-human CD18, clone 6.7; cat. no. 555924 (BD Biosciences, USA) (5 µg/mL); application, FACS analysis.
Anti-human CD18, clone TS1/18; cat. no. 302108 (BioLegend, USA) (5 µg/mL); application, FACS analysis.
Anti-human CD32, clone IV.3; cat. no. GTX-14572 (Gene Tex, USA) (5 µg/mL); application, FACS and functional analysis.
Anti-human CD41/CD61, clone A2A9/6; cat. no. 359810 (BioLegend, USA) (5 µg/mL); application, FACS analysis.
Anti-human CD44, clone G44-26; cat. no. 555478 (BD Biosciences, USA) (5 µg/mL); application, FACS analysis.
Anti-human CD51, clone NKI-M9; cat. no. 565835 (BD Biosciences, USA) (5 µg/mL); application, FACS analysis.
Anti-human CD54, clone HA58; cat. no. 559771 (BD Biosciences, USA) (5 µg/mL); application, FACS analysis.
Anti-human CD55, clone BRIC 216; cat. no. sc-59092 (Santa Cruz, USA) (5 µg/mL); application, FACS, WB, and functional analysis.
Anti-human CXCR1, clone 5A12; cat. no. 555939 (BD Biosciences, USA) (5 µg/mL); application, FACS analysis.
Anti-human CXCR4, clone 12G5; cat. no. 555974 (BD Biosciences, USA) (5 µg/mL); application, FACS analysis.
Anti-human CD62L, clone DREG-56; cat. no. 555544 (BD Biosciences, USA) (5 µg/mL); application, FACS analysis.

Anti-human CD62E, clone 68-5H11; cat. no. 551144 (BD Biosciences, USA) (5 µg/mL); application, FACS analysis.
Anti-human CD62P, clone AK4; cat. no. 304906 (BioLegend, USA) (5 µg/mL); application, FACS analysis.
Anti-human CD63, clone H5C6; cat. no. 557288 (BD Biosciences, USA) (5 µg/mL); application, FACS analysis.
Anti-human CD66b, clone G10F5; cat. no. 561645 (BD Biosciences, USA) (5 µg/mL); application, FACS analysis.
Anti-human CD66b-PerCP-Cy5.5, clone G10F5; cat. no.305108 (Biolegend, USA) (5 µg/mL); application, FACS sorting.
Anti-human CD177, clone MEM-166; cat. no. 315802, 315806 (BioLegend, USA) (5 µg/mL); application, FACS and WB analysis.
Anti-human CD177, clone C5; cat. no. sc-376329 (Santa Cruz, USA) (1:500); application, FACS and PLA analysis.
Anti-human thrombin R, clone ATAP2; cat. no. sc-13503 (Santa Cruz, USA) (5 µg/mL); application, FACS analysis.
Anti-human PR3, clone MCPR3-2; cat. no. MA-11945 (Thermo Fisher, USA) (5 µg/mL); application, FACS, ELISA, and WB analysis.
Anti-human PR3, clone CLB-12.8 (Sanquin, the Netherlands) (5 µg/mL); application, FACS and functional analysis.
Anti-human PR3, clone 4A5, (EURO DIAGNOSTICA, Sweden) (2 µL/test); application, FACS analysis.
Anti-human OLFM4, polyclonal; cat. no. ab85046 (Abcam, USA) (5 µg/mL); application, FACS analysis.
Anti-human PAR2, clone SAM11 cat. no. sc-13504 (Santa Cruz, USA) (5 µg/mL); application, FACS, PLA, WB, and functional blocking analysis.
Anti-human PAR2, clone MAB3949; cat. no. 344222 (R&D, USA) (5 µg/mL); application, FACS and functional blocking analysis.
Anti-human EPCR, clone RCR-49; cat. no. sc-53982 (Santa Cruz, USA) (5 µg/mL); application, FACS analysis.
Anti-human VCAM-1, clone 51-10C9; cat. no. 561679 (BD Biosciences, USA) (5 µg/mL); application, FACS analysis.
Anti-human EMR2, clone 2A1; cat. no. MA5-16474 (Invitrogen, USA) (5 µg/mL); application, FACS and functional analysis.
Anti-human GPR97, clone G97-A; in house. (5 µg/mL); application, FACS, WB, PLA, and functional blocking analysis.
Anti-human GPR97, clone BGP; in house. (5 µg/mL); application, FACS and functional blocking analysis.
AffiniPure Goat Anti-Rabbit IgG (H+L)(FITC), polyclonal; cat. no. 111-095-144 (Jackson ImmunnoResearch, USA) (1:200); application, FACS and PLA analysis.
Goat anti-mouse IgG (Alexa Fluor 647), polyclonal; cat. no. A-21235 (Thermo Fisher, USA) (1:400); application, FACS and PLA analysis.
Normal mouse IgG1-APC, cat. no. sc-2888 (Santa Cruz, USA) (5 µg/mL); application, FACS analysis.
Normal mouse IgG1-PE, cat. no. sc-2866 (Santa Cruz, USA) (5 µg/mL); application, FACS analysis.
(HRP)-conjugated goat anti-mouse Fc, polyclonal; cat. no.305108 (Sigma-aldrich, USA) (1:2000); application, WB analysis.
goat anti-mouse kappa chain HRP, polyclonal ; cat. no. AP200P (Sigma-aldrich, USA) (1 µg/mL); application, WB analysis.
anti-human eNOS, Clone 3/eNOS/NOS Type III; cat. no. 560103 (BD, USA) (5 µg/mL); application, FACS analysis.

Validation

All commercially available primary antibodies against human CD markers and specific receptors used in the study were well-validated for the detection of human leukocyte antigens in flow cytometry application as described on the manufacturers' websites.
Anti-human CD11a antibody (clone G43-25B; cat. no. 555379); validation: Product website (BD Biosciences) https://www.bdbiosciences.com/en-au/products/reagents/flow-cytometry-reagents/research-reagents/single-color-antibodies-ruo/pe-mouse-anti-human-cd11a.555380
Anti-human CD11b antibody (clone ICRF44; cat. no. 555388); validation: Product website (BD Biosciences) https://www.bdbiosciences.com/en-au/products/reagents/flow-cytometry-reagents/research-reagents/single-color-antibodies-ruo/pe-mouse-anti-human-cd11b.555388
Anti-human CD16 antibody (clone 3G8; cat. no. 555407); validation: Product website (BD Biosciences) https://www.bdbiosciences.com/ko-kr/products/reagents/flow-cytometry-reagents/research-reagents/single-color-antibodies-ruo/PE-Mouse-Anti-Human-CD16.555407
Anti-human CD16 antibody (clone 3G8; cat. no. 302002); validation: Product website (BioLegend) https://www.biolegend.com/fr-lu/products/purified-anti-human-cd16-antibody-571
Anti-human CD16b-FITC (clone REA589; cat. no. 130-126-529); validation: Product website (Miltenyi Biotec) https://www.miltenyibiotec.com/UN-en/products/cd16b-antibody-anti-human-reafinity-rea589.html?countryRedirected=1#gref
Anti-human CD18 antibody (clone 6.7; cat. no. 555924); validation: Product website (BD Biosciences) https://www.bdbiosciences.com/en-ca/products/reagents/flow-cytometry-reagents/research-reagents/single-color-antibodies-ruo/pe-mouse-anti-human-cd18.555924
Anti-human CD18 antibody (clone TS1/18; cat. no. 302108) Anti-human CD32, clone IV.3; cat. no. GTX-14572 (Gene Tex (BioLegend) https://www.biolegend.com/fr-ch/products/purified-anti-human-cd18-antibody-851
Anti-human CD32 antibody (clone IV.3; cat. no. GTX-14572); validation: Product website (Gene Tex) https://www.genetex.com/Product/Detail/CD32-antibody-IV-3/GTX14572
Anti-human CD41/CD61 antibody (clone A2A9/6; cat. no. 359810); validation: Product website (BioLegend) https://www.biolegend.com/fr-lu/products/purified-anti-human-cd41-cd61-antibody-8856?Clone=A2A9/6
Anti-human CD44 antibody (clone G44-26; cat. no. 555478); validation: Product website (BD Biosciences) https://www.bdbiosciences.com/ko-kr/products/reagents/flow-cytometry-reagents/research-reagents/single-color-antibodies-ruo/fitc-mouse-anti-human-cd44.555478
Anti-human CD51 antibody (clone NKI-M9; cat. no. 565835); validation: Product website (BD Biosciences) https://www.bdbiosciences.com/en-ca/products/reagents/flow-cytometry-reagents/research-reagents/single-color-antibodies-ruo/alexa-fluor-647-mouse-anti-human-cd51.565835
Anti-human CD54 antibody (clone HA58; cat. no. 559771); validation: Product website (BD Biosciences) https://www.bdbiosciences.com/en-us/products/reagents/flow-cytometry-reagents/research-reagents/single-color-antibodies-ruo/apc-mouse-anti-human-cd54.559771
Anti-human CD55 antibody (clone BRIC 216; cat. no. sc-59092); validation: Product website (Santa Cruz) https://www.scbt.com/p/cd55-antibody-bric-216
Anti-human CXCR1 antibody (clone 5A12; cat. no. 555939); validation: Product website (BD Biosciences) https://www.bdbiosciences.com/en-au/products/reagents/flow-cytometry-reagents/research-reagents/single-color-antibodies-ruo/fitc-mouse-anti-human-cd181.555939
Anti-human CXCR4 antibody (clone 12G5; cat. no. 555974); validation: Product website (BD Biosciences) https://www.bdbiosciences.com/en-us/products/reagents/flow-cytometry-reagents/research-reagents/single-color-antibodies-ruo/pe-mouse-anti-human-cd184.555974
Anti-human CD62L antibody (clone DREG-56; cat. no. 555544); validation: Product website (BD Biosciences) https://www.bdbiosciences.com/en-at/products/reagents/flow-cytometry-reagents/research-reagents/single-color-antibodies-ruo/pe-mouse-anti-human-cd62l.555544
Anti-human CD62E antibody (clone 68-5H11; cat. no. 551144); validation: Product website (BD Biosciences) https://www.bdbiosciences.com/en-us/products/reagents/flow-cytometry-reagents/research-reagents/single-color-antibodies-ruo/apc-mouse-anti-human-cd62e.551144/

Anti-human CD62P antibody (clone AK4; cat. no. 304906); validation: Product website (BioLegend) https://www.biolegend.com/fr-fr/products/pe-anti-human-cd62p-p-selectin-antibody-595
Anti-human CD63 antibody (clone H5C6; cat. no. 557288); validation: Product website (BD Biosciences) https://www.bdbiosciences.com/en-au/products/reagents/flow-cytometry-reagents/research-reagents/single-color-antibodies-ruo/fitc-mouse-anti-human-cd63.557288
Anti-human CD66b antibody (clone G10F5; cat. no. 561645); validation: Product website (BD Biosciences) https://www.bdbiosciences.com/en-tw/products/reagents/flow-cytometry-reagents/research-reagents/single-color-antibodies-ruo/alexa-fluor-647-mouse-anti-human-cd66b.561645
Anti-human CD66b-PerCP-Cy5.5 (clone G10F5; cat. no.305108); validation: Product website (Biolegend) https://www.biolegend.com/de-de/search-results/percp-cyanine5-5-anti-human-cd66b-antibody-6585
Anti-human CD177 antibody (clone MEM-166; cat. no. 315802, 315806); validation: Product website (BioLegend) https://www.biolegend.com/it-it/products/purified-anti-human-cd177-antibody-2791
Anti-human CD177 antibody (clone C5; cat. no. sc-376329); validation: PLoS Genet 2015; 11(5): e1005255. doi: 10.1371/journal.pgen.1005255
Anti-human thrombin R antibody (clone ATAP2; cat. no. sc-13503); validation: Product website (Santa Cruz) https://www.scbt.com/p/thrombin-r-antibody-atap2
Anti-human PR3 antibody (clone MCPR3-2; cat. no. MA-11945); validation: Product website (Thermo Fisher) https://www.thermofisher.com/antibody/product/PR3-Antibody-clone-MCPR3-2-Monoclonal/MA5-11945
Anti-human PR3 antibody, clone CLB-12.8 (Sanquin, the Netherlands); validation: J. Immunol. 2005,174:6381-6390. doi.org/10.4049/jimmunol.174.10.6381
Anti-human PR3 antibody, clone 4A5, (EURO DIAGNOSTICA, Sweden); validation: Clin. Exp. Immunol. 2004; 138: 183–192. doi.org/10.1111/j.1365-2249.2004.02566.x
Anti-human OLFM4 antibody (polyclonal; cat. no. ab85046); validation: Product website (Abcam) https://www.abcam.com/olfm4-antibody-ab85046.html
Anti-human PAR2 antibody (clone SAM11 cat. no. sc-13504); validation: Product website (Santa Cruz) https://www.scbt.com/p/par-2-antibody-sam11
Anti-human PAR2 antibody (clone MAB3949; cat. no. 344222); validation: Product website (R&D) https://www.rndsystems.com/products/human-par2-antibody-344222_mab3949
Anti-human EPCR antibody (clone RCR-49; cat. no. sc-53982); validation: Product website (Santa Cruz) https://www.scbt.com/p/epcr-antibody-rcr-49
Anti-human VCAM-1 antibody (clone 51-10C9; cat. no. 561679); validation: Product website (BD Biosciences) https://www.bdbiosciences.com/en-au/products/reagents/flow-cytometry-reagents/research-reagents/single-color-antibodies-ruo/pe-mouse-anti-human-cd106.561679
Anti-human EMR2 antibody (clone 2A1; cat. no. MA5-16474); validation: Product website (Invitrogen) https://www.fishersci.com/shop/products/anti-cd312-clone-2a1/PIMA516474
AffiniPure Goat Anti-Rabbit IgG (H+L)(FITC) antibody (polyclonal; cat. no. 111-095-144); validation: Product website (Jackson ImmunnoResearch) https://www.jacksonimmuno.com/catalog/products/111-095-144
Goat anti-mouse IgG (Alexa Fluor 647) antibody (polyclonal; cat. no. A-21235); validation: Product website (Thermo Fisher) https://www.thermofisher.com/antibody/product/Goat-anti-Mouse-IgG-H-L-Cross-Adsorbed-Secondary-Antibody-Polyclonal/A-21235
Normal mouse IgG1-APC (cat. no. sc-2888); validation: Product website (Santa Cruz) https://www.scbt.com/p/normal-mouse-igg1-apc
Normal mouse IgG1-PE (cat. no. sc-2866); validation: Product website (Santa Cruz) https://www.scbt.com/p/normal-mouse-igg1-pe
(HRP)-conjugated goat anti-mouse Fc (polyclonal; cat. no.305108); validation: Product website (Sigma-aldrich) https://www.sigmaaldrich.com/TW/en/product/mm/12349
Goat anti-mouse kappa chain HRP (polyclonal ; cat. no. AP200P); validation: Product website (Sigma-aldrich) https://www.sigmaaldrich.com/TW/en/product/mm/ap200p
anti-human eNOS, Clone 3/eNOS/NOS Type III antibody (cat. no. 560103); validation: Product website (BD Biosciences) https://www.bdbiosciences.com/en-us/products/reagents/flow-cytometry-reagents/research-reagents/single-color-antibodies-ruo/pe-mouse-anti-enos.560103
Anti-human PR3 Ab, clone MCPR3-2; validation: Clinical & Experimental Immunology, 1999; 118(3):487-496. (DOI:10.1046/j.1365-2249.1999.01079.x)
Anti-human PR3 Ab, clone 4A5; validation: JOURNAL OF BIOLOGICAL CHEMISTRY 2008; 283:35976–35982. (doi.org/10.1074/jbc.M806754200)
Anti-human PR3 Ab, clone CLB12.8; validation: Clinical & Experimental Immunology, 1999; 118(3):487-496. (DOI:10.1046/j.1365-2249.1999.01079.x)
Anti-human PAR2 Ab, clone SAM11; validation: Mol Cancer Res 2004; 2:395-402. (DOI:10.1158/1541-7786.395.2.7)
Anti-human PAR2 Ab, clone MAB3949; validation: Thrombosis Research 2019; 177:91-101. (doi.org/10.1016/j.thromres.2019.02.032).
In house anti-GPR97 mAbs were validated for the use of WB and IF studies and described in our previous publication (Hsiao et al, 2018 Frontiers in Immunology 9: 2830, PMID: 30559745).

Eukaryotic cell lines

Policy information about cell lines

Cell line source(s)

HT1080 cell (CCL-121™, American Type Culture Collection)
NIH/3T3 cell (CRL-1658™, American Type Culture Collection)
HeLa cell (CCL-2™, American Type Culture Collection)
M059K cell (CRL-2365™, American Type Culture Collection)
U-87 MG (HTB-14™, American Type Culture Collection)
C32 cell (CRL-1585™, American Type Culture Collection)
COS-7 cell (CRL-1651™, American Type Culture Collection)
MCF-7 cell (CRL-3435™, American Type Culture Collection)
HEK-293T cell (CRL-3216™, American Type Culture Collection)
K-562 cell (CCL-243™, American Type Culture Collection)

	A2058 cell (CRL-11147™, American Type Culture Collection)<br>A375 cell (CRL-1619™, American Type Culture Collection)<br>Jurkat cell (TIB-152™, American Type Culture Collection)<br>CHO-K1 cell (CCL-61™, American Type Culture Collection)<br>MeWo cell (HTB-65™, American Type Culture Collection)<br>MEL-14 cell (HB-132™, American Type Culture Collection)<br>THP-1 cell (TIB-202™, American Type Culture Collection)<br>U-937 cell (CRL-1593.2™, American Type Culture Collection)<br>Chang Liver cell (CCL-13™, American Type Culture Collection)<br>Hep-3B cell (HB-8064™, American Type Culture Collection)<br>Hep-G2 cell (HB-8065™, American Type Culture Collection)<br>SK-Hep cell (HTB-52™, American Type Culture Collection)<br>SK-MEL5 cell (HTB-70™, American Type Culture Collection)<br>RPMI-7951 cell (HTB-66™, American Type Culture Collection)<br>HL-60 cell (CCL-240™, American Type Culture Collection)<br>Raw264.7 cell (TIB-71™, American Type Culture Collection)<br>HUVEC cell (H-UV001, Bioresource Collection and Research Center, Taiwan)<br>G5T/VGH cell (60194, Bioresource Collection and Research Center, Taiwan)
Authentication	Morphology analysis with microscopy was used for cell line authentication.
Mycoplasma contamination	All cell lines were tested negative for mycoplasma contamination.
Commonly misidentified lines<br>(See ICLAC register)	No misidentified cell lines were used in the study.

Human research participants

Policy information about studies involving human research participants

Population characteristics	Donors of normal blood samples- female and male healthy volunteer blood donors, no history of chronic disease, 20-60 years old. Donors of diseased blood samples (sepsis, GPA, and MPA) - female and male donors admitted for outpatient clinics, 20-60 years old.
Recruitment	Participants were recruited either by posters displayed in public or from out-patient clinics. Participants of the study comprised individuals diagnosed with bacteria sepsis, MPA, and GPA. Control participants included healthy individuals. Characteristics of patients recruited into the study are listed in the Source Data file. Healthy individuals were recruited to exclude the impact of any chronic health condition. Donors of both genders were recruited to avoid misleading conclusions that could refer only to one gender. In each experiment male and female blood cells were tested and the male and female group sizes were equal. The patients were recruited according to the objective criteria of age, gender and health status and no self-selection bias affected the recruitment.
Ethics oversight	All blood donors gave informed consent and all experimental protocols were approved by Chang Gung Memorial Hospital Ethics Committee (CGMH IRB No: 201701852B0, 201901358B0, 201901293B0, and 202002255B0)

Note that full information on the approval of the study protocol must also be provided in the manuscript.

Flow Cytometry

Plots

Confirm that:

☒ The axis labels state the marker and fluorochrome used (e.g. CD4-FITC).

☒ The axis scales are clearly visible. Include numbers along axes only for bottom left plot of group (a 'group' is an analysis of identical markers).

☒ All plots are contour plots with outliers or pseudocolor plots.

☒ A numerical value for number of cells or percentage (with statistics) is provided.

Methodology

Sample preparation	Human neutrophils were isolated from fresh venous blood donated by healthy male and female volunteers and diseased patients. Blood samples were drawn into the collection tubes coated with sodium heparin and neutrophil isolation was performed using PolymorphprepTM density gradient centrifugation (Axis-Shield, Oslo, Norway). For the isolation of murine neutrophils, blood was collected by cardiac puncture and separated by Ficoll (GE Healthcare) gradient separation. Following the lysis of the remnant erythrocytes, isolated neutrophils were checked for purity by flow cytometry with specific surface marker (CD66b) and resuspended in RPMI medium containing 10% fetal bovine serum (FBS) for all following experiments unless otherwise denoted. The isolation procedure routinely produced a >95% pure and viable neutrophil population. When indicated, isolated human neutrophils were further subjected to the magnetic cell sorting (MACS) separation of CD177+ and CD177- sub-populations using PE-conjugated anti-CD177 Ab coupled to the MACS MicroBeads and Separator (Miltenyi Biotec, Bergisch Gladbach, Germany) according to the manufacturer's recommendations.
Instrument	FACSCanto II, FACSCalibur, and FACSAriaTM II flow cytometer (BD Biosciences)

Software	BD CellQuest Pro Version 6.0 for data collection and FlowJo v7.6.1 for data analysis
Cell population abundance	For human neutrophil FACS analysis, at least a million events were acquired. The purity of isolated neutrophils was determined by FACS staining of CD66b and routinely to be more than 95%.
Gating strategy	The gating strategy of neutrophils from participants for studies in Figure 1 was described in the Supporting information file using FSC vs. SSC followed by CD16b staining.

☒ Tick this box to confirm that a figure exemplifying the gating strategy is provided in the Supplementary Information.
